# Transient hexagonal structures in sheared emulsions of isotropic inclusions on smectic bubbles in microgravity conditions

**DOI:** 10.1038/s41598-021-98166-7

**Published:** 2021-09-27

**Authors:** P. V. Dolganov, N. S. Shuravin, V. K. Dolganov, E. I. Kats, R. Stannarius, K. Harth, T. Trittel, C. S. Park, J. E. Maclennan

**Affiliations:** 1 Institute of Solid State Physics, Russian Academy of Sciences (ISSP RAS), 142432 Chernogolovka, Moscow Region, Russia; 2 L.D. Landau Institute for Theoretical Physics, Russian Academy of Sciences, 142432 Chernogolovka, Moscow Region, Russia; 3 Institute of Physics, Otto von Guericke University, 39106 Magdeburg, Germany; 4 Department of Physics, University of Colorado Boulder, Boulder, CO 80309 USA

**Keywords:** Condensed-matter physics, Surfaces, interfaces and thin films, Fluid dynamics

## Abstract

We describe the collective behavior of isotropic droplets dispersed over a spherical smectic bubble, observed under microgravity conditions on the International Space Station (ISS). We find that droplets can form two-dimensional hexagonal structures changing with time. Our analysis indicates the possibility of spatial and temporal periodicity of such structures of droplets. Quantitative analysis of the hexagonal structure including the first three coordination circles was performed. A peculiar periodic-in-time ordering of the droplets, related to one-dimensional motion of droplets with non-uniform velocity, was found.

## Introduction

Self-organization of embedded particles is one of the richest and most intriguing phenomena in the physics of free-standing films of liquid crystals. Self-organization was discovered and has been widely investigated in polar Smectic-C* (SmC*), nonpolar Smectic-*C* (Sm*C*) and Smectic-*A* (Sm*A*) films^1–10^ and is associated with deformations of the orientational ordering of molecules in smectic layers. Topological defects of the director field localized near the particles or on their boundary play an essential role in self-organization^11^. At large distances, the embedded particles interact as topological dipoles or quadrupoles. The distance between the surfaces of the particles in the observed structures (linear and branched chains) is typically less than the particle diameter in the case of both dipole–dipole and quadrupole–quadrupole interactions. At present, the mechanism of self-organization involving topological defects is well understood, with the collective behavior of particles described by continuum theory using analytical and numerical calculations. However, for a large number of particles in Sm*C* free standing films, qualitatively different structures are observed with hexagonal ordering and with interparticle distances considerably larger than for the case of quadrupole–quadrupole and dipole–dipole interaction^6,12^. The mechanism of formation of two-dimensional hexagonal structures is not yet fully understood.

In this work, we analyze the static and dynamic properties of the self-organized particles (droplets of isotropic liquid) leading to the formation of hexagonal structures in smectic *A* (Sm*A*) films. In contrast to the smectic *C* phase, the molecules are oriented preferentially normal to the smectic layers, so that there are no topological dipoles or quadrupoles and elastic interactions via in-plane orientational field (**c**-director) can be excluded. A spherical film geometry was chosen in order to minimize the influence of the film meniscus on objects in the film, and microgravity was needed to prevent the inclusions from sedimenting in the gravitation field. The experimental data analyzed in our paper were obtained in microgravity conditions (< 10^–3^ g) on the International Space Station (ISS)^1,2^ as a part of the OASIS mission. The data were transported from the ISS and transferred to Boulder (USA), Magdeburg (Germany), and Chernogolovka (Russia).Various types of collective droplet behavior were found. Results published by the Boulder and Magdeburg groups^1,2^ motivated us to analyze the positional and orientational ordering of droplets in the hexagonal structure. The widths of the distribution functions for radial and azimuthal droplet positions increase with distance. In the presence of velocity gradients in the film, a periodic appearance and disappearance of the hexagonal ordering of the droplets was observed.

Such non-trivial dynamic behavior suggests to look for some analogy to the concept of time crystals proposed by F. Wilczek in 2012^13,14^ and coming back into fashion with a number of high-impact publications. Although the original idea of F. Wilczek has been proven as impossible^15^, time crystals are still the subject of vigorous scientific debate (see, for example^16^). Wilczek’s theory has been replaced by a less fanciful definition of a time crystal, for a driven system with time-translation broken by integer multiples of the driving period (as evidenced by observation of a subharmonic response). There exist many examples of systems displaying various types of spatio-temporal order. To name a few, we mention classical pattern formation dynamic instabilities (ranging from the well-known Belousov–Zhabotinsky chemical reactions to electro-convective instabilities in nematic liquid crystals, see, for example^17^). Very recently, another dynamic analogue of time crystalline order has been studied^18,19^. As is often the case, the abundance of terminology seen in the literature (dynamic pattern formation, discrete time crystals, Floquet time crystals) reflects the complexity of the phenomenon. The possibility of forming a time crystal is closely connected with the fundamental concept of spontaneous symmetry breaking used in different areas of physics, ranging from conventional condensed matter phase transitions to the cosmological Kibble mechanism of topological defect formation^20^. It has been shown that discrete time crystals can exist only in non-equilibrium systems^21–23^ and they have been realized in several quantum systems^24,25^. In this paper we analyze the spatial and temporal distribution of inclusions in smectic films as observed in the OASIS experiment.

## Results

### Materials and experimental setup

**Figure 1:**
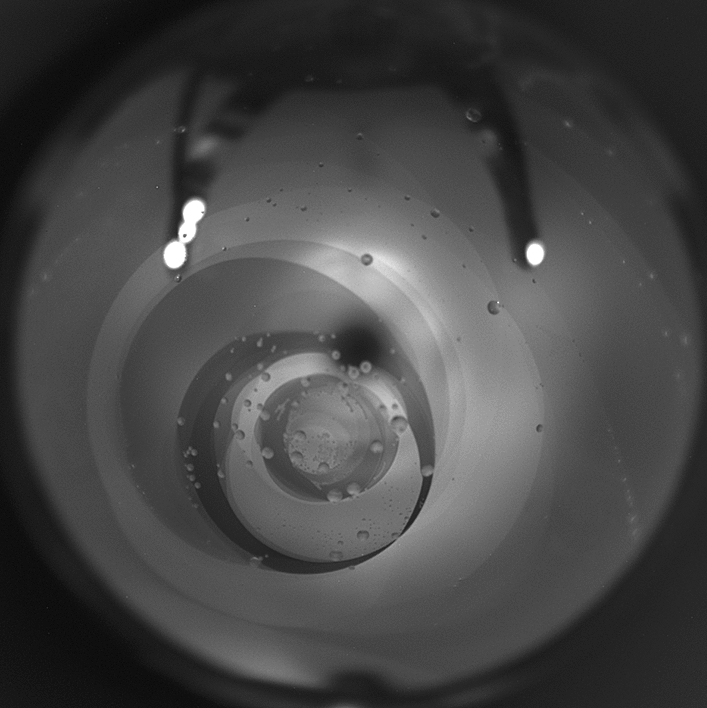
Smectic film in the form of a spherical bubble viewed in reflected white light. Regions of the film with different thickness have different brightness. Regions close to the center of the image have larger thickness than the outer regions. The bubble is heated close to the temperature of the Sm*A*‒Iso phase transition. Large droplets of the isotropic phase are visible in this image; small droplets can be seen in the micro images (Figs. 2, 5). The diameter of the bubble is about 1.5 cm.

In the experiment, a Sm*A* liquid crystal (Displaytech MX12160) with a direct phase transition from the Sm*A* to the isotropic (Iso) phase (Sm*A*– 51.1 °C—Iso) was employed^1,2^. The method used to prepare the smectic bubbles with diameter about 1.5 cm is described in^1^. Two video cameras were used, one to capture images of the whole bubble (Fig. 1) and the other to record, at higher magnification, the structures formed by droplets on the film. The bubble had regions of uniform thickness, on which the observation of micro images was made. Images were captured at a frame rate of 30 fps.

**Figure 2:**
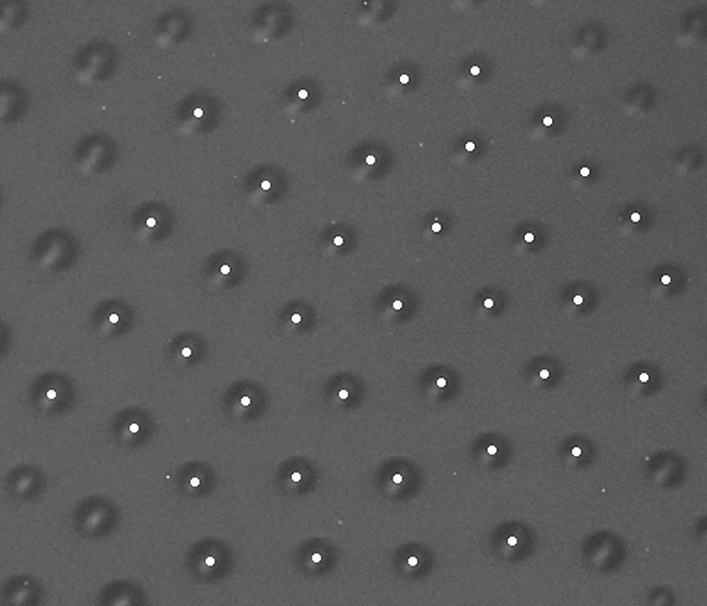
Droplets with hexagonal ordering in a film region of uniform thickness. The structure is conserved in time. White dots show the droplet centers. The horizontal size of the image is 230 μm.

On heating close to the temperature of the Sm*A*‒Iso transition (actually, slightly above the bulk transition temperature), droplets of isotropic phase formed in the film. Droplet sizes and their number density can differ in different regions of the bubble, which are separated by dislocation lines. The registration of micro-images of groups of droplets was performed with droplets of about 15 μm in diameter. In some regions of the film where the number density of droplets was greater than around 500/mm^2^, a quasi-hexagonal structure of droplets could be observed (Fig. 2)^2^. The structure resembles the hexagonal ordering of droplets observed earlier in flat free-standing smectic C films under terrestrial conditions^6,12^. The distances between droplets in the hexagonal structure can be significantly larger than the droplet size (Fig. 2). The nature of the long-range interactions stabilizing this structure is not clear. As mentioned above, in Sm*A* films there are no in-plane interactions related to the **c**-director, which stabilize ordered structures of inclusions in films of tilted smectics^6,12^. The smectic C droplet arrangements are characterized by a fixed lattice distance. In smectic A, capillary forces, interactions due to smectic elasticity, van der Waals interactions, or critical fluctuations of the smectic order parameter could be relevant in the vicinity of the phase transition. The hexagonal structure is stabilized by repulsive interactions of the droplets, which are constrained to certain film regions. In the hexagonal lattice, the droplets maximize their mutual distances at a given number density in the film plane. One can consider these structures as quasi-two-dimensional colloidal crystals^2^. The difference to previously described colloidal crystals formed by particles at the surface of a liquid is that in the smectic film system there is no bulk substrate.

### Experimental data analysis

**Figure 3:**
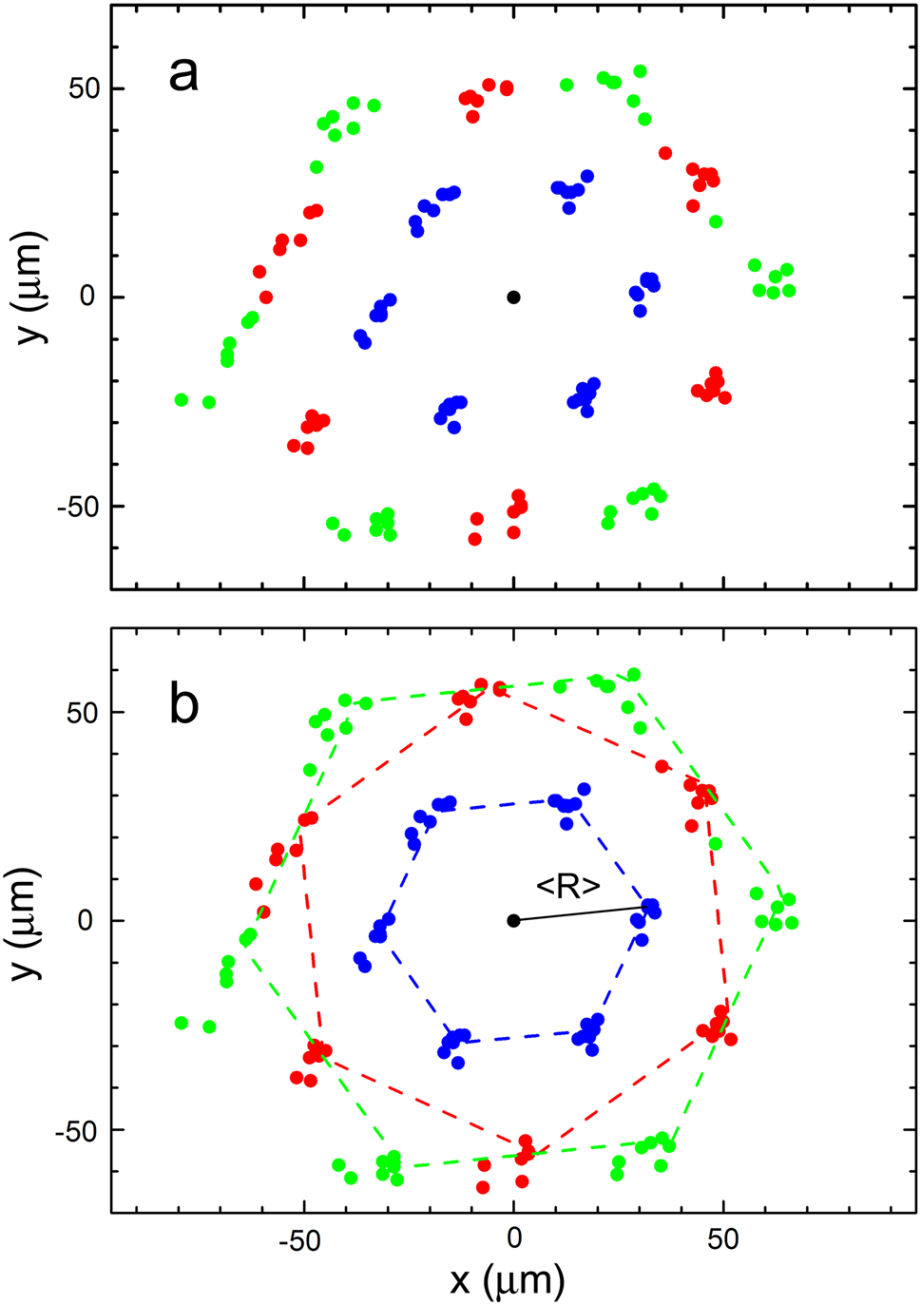
(**a**) Spatial arrangement of droplets in the first three coordination circles determined directly from the image of Fig. 2. Each group of seven dots was obtained for the same directions between droplets. (**b**) Spatial arrangement of droplets corrected to account for image distortion in Fig. 2. Dashed lines show symmetrical hexagons whose vertex coordinates correspond to minimal summary distances from the droplet positions. The average distance $\left\langle R \right\rangle$ between droplets in the first coordination circle is 32 ± 2 μm.

First, we analyze the ordering of droplets in the hexagonal structure. Figure 3a illustrates the spatial arrangement of the droplets extracted from the photo of Fig. 2. Our analysis of the structures was performed for droplets with their centers located in the first three coordination circles with mean radius from the central droplet $R = a, \sqrt 3 a, 2a$, where *a* is the distance between nearest droplets. Positions of the droplets in the first three coordination circles are plotted (Fig. 3) with respect to the origin. Measurements were performed on droplets which are marked in Fig. 2 by white dots. Because the bubble surface is curved, only part of the micro image is in the focal plane of the camera. This induces a slight distortion in the image, namely shrinkage of the distance in the direction parallel to the inclination plane. As a result of this optical aberration, the droplets at one of the edges of the original images are out of focus. A correction procedure was applied to the data in Fig. 3a: the image was elongated in the direction which was chosen to minimize the dispersion of droplet distances in the first coordination circle, i.e., to minimize the sum of squared deviations of the distance between droplets from the average interparticle distance $\left\langle R \right\rangle$. We are confident that after this procedure the distortion due to tilt of the film plane with respect to the camera is corrected. The correction factor was typically of the order of 10%.

**Figure 4:**
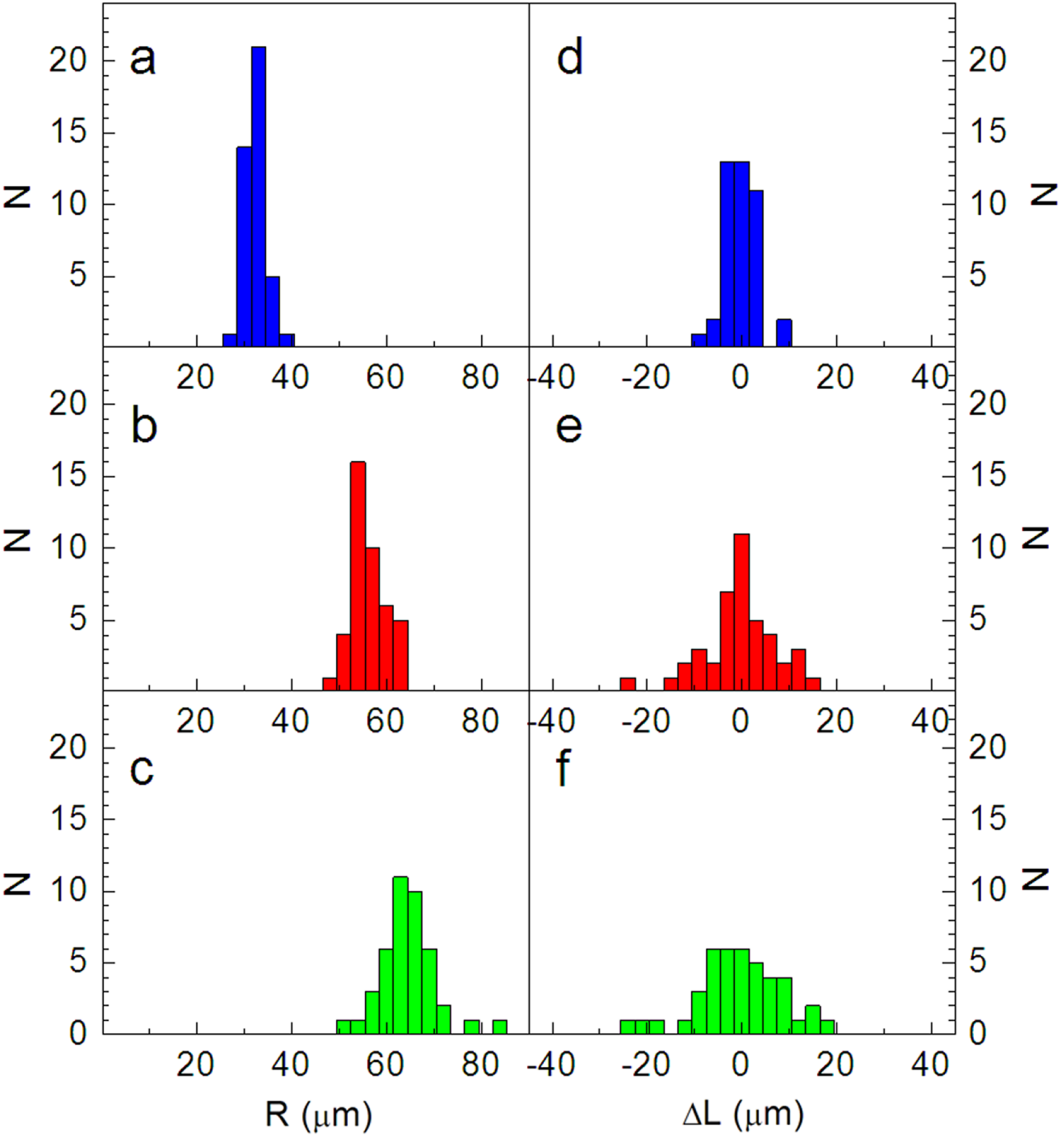
(**a**–**c**) Radial distributions *N*(*R*) of the droplet positions in the first (a), second (b) and third (**c**) coordination circles. (**d**–**f**) Azimuthal distributions *N*(Δ*L*) of the droplet positions in the first (**d**), second (**e**) and third (**f**) coordination circles. The histograms are plotted using the spatial distribution of droplets in Fig. 3b.

The distribution of droplets in the three coordination circles after correction is shown in Fig. 3b. Dashed lines show symmetrical hexagons plotted so that the positions of their vertices correspond to the minimal sum of the distances from droplet positions in the corresponding group. The orientational and positional distribution of the droplets in the lattice is quantitatively described in Fig. 4. The image gives histograms of the radial (a-c) and azimuthal (d-f) distribution of droplets in the first (a,d), second (b,e) and third (c,f) coordination circles. The distribution of azimuthal positions measured normal to the directions of the main diagonals of hexagons in Fig. 3b is plotted in (d–f). The half-widths of the radial distributions in the second and third coordination circles increase with respect to the first circle. The widths of the azimuthal distributions in Fig. 4e,f also increase considerably with respect to the first coordination circle. The azimuthal distributions as a whole are wider than the corresponding radial ones. The distribution of positional and orientational ordering in the structure is distinct from structures with bond orientational order (see, for example^26,27^). Two-dimensional hexatic ordering is characterized by a quasi-long-range bond-orientational order, while the positional order is only short range. Phases with hexatic order have been found in several systems, such as electrons at the surface of helium, dust plasmas, charged polymer colloids, and smectic liquid crystals^28–31^.

Similarly to equilibrium systems, it is possible to introduce an order parameter describing the two-dimensional, quasi-hexagonal ordering of droplets in smectic films. This would involve finding the dynamic action derived from the non-linear dynamic equations. This challenging theoretical task is beyond the scope of our study.

**Figure 5:**
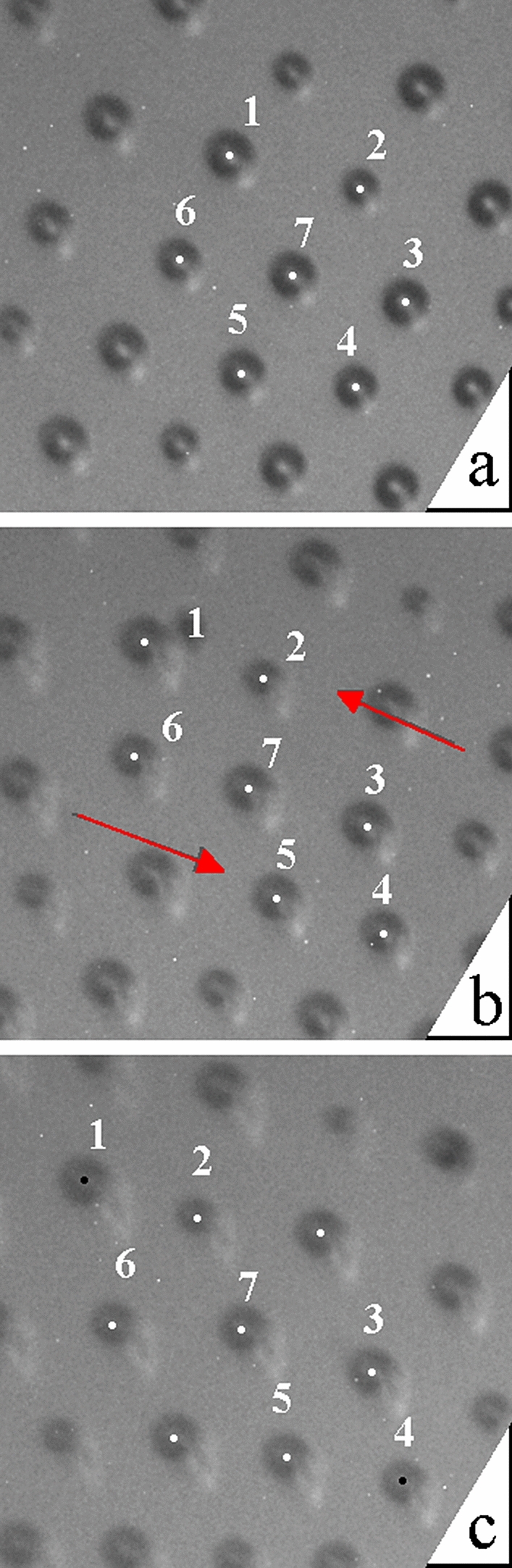
Hexagonal ordering is exhibited by droplets in the smectic film at certain moments of time. The droplets denoted 1, 2 and 4, 5 move in opposite directions relative to the row of droplets 3, 6, and 7. Hexagonal ordering (**a**) is destroyed (**b**) and then reconstructed after some time (**c**). Frame (**b**) is taken 55 s after frame (**a**), and frame (**c**) 40 s later. The horizontal size of the images is 191 μm.

**Figure 6:**
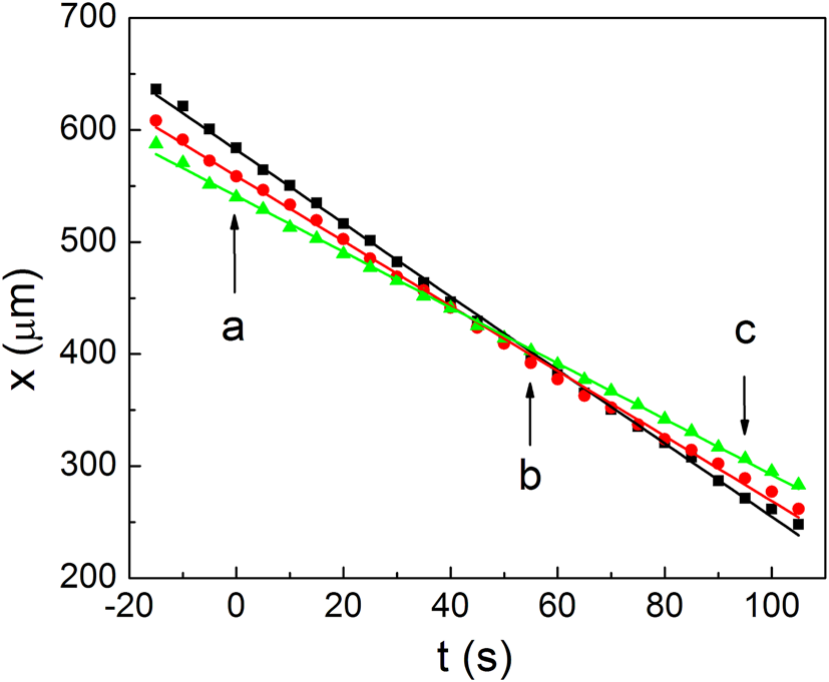
Temporal dependence of the x-coordinate of three droplets in Fig. 5: droplet 2 (squares), droplet 7 (circles) and droplet 5 (triangles). Moments corresponding to frames (a), (b) and (c) in Fig. 5 are indicated by arrows.

Earlier work by Klopp et al.^2^ focused on lattices that were either stationary or moved within a uniformly flowing film region without spatial velocity gradients. Here, we focus on such parts of the lattice that are exposed to shear flow. In our case, close to the temperature of the Sm*A*-Iso transition, regions of different thickness (Fig. 1) can transform and change their shape. As a result, the material of the film and the droplets also move. In the case of a uniform drift of the droplets in the film plane, the hexagonal structure is conserved. Interesting effects were observed when the velocity of the droplets was non-uniform. In Fig. 5a, the droplets move nearly along one of the longer diagonals of the hexagon, with a velocity gradient perpendicular to that direction. To facilitate tracking the droplets, the droplet centers in Fig. 5 which form a hexagon are marked by white dots. Figure 6 shows the dependence of the horizontal coordinate of three of the droplets in Fig. 5 (namely, droplets 2, 7 and 5) on time. The average horizontal velocity of the droplets in Fig. 5 is about 3 μm/s. However, the droplets have different velocities, as is evident from Fig. 6. The velocity of droplets above and below the hexagon is larger and smaller, respectively, than the average drift velocity at its center by about 0.4 μm/s. This shearing motion leads to destruction of the original hexagonal structure (Fig. 5b) and its reappearance after some time (Fig. 5c) when the droplets in the upper and lower row shift by about one lattice parameter (average interparticle distance) in opposite directions. The structure formed by droplets in which the hexagonal order disappears and reappears resembles (but is not identical with) a time crystal. In our observation, a nearly constant gradient of the mean velocity was created only in the three adjacent rows of droplets. It is worth noting that if a constant velocity gradient were created in a larger number of rows, one could expect much richer behavior, e.g., the appearance of a line of hexagons perpendicular to the motion of droplets at discrete moments of time (which are determined by the velocity of the row of hexagons with the smallest velocity). A process similar to the one described above can be observed in shear melting of three-dimensional colloidal crystals, where a so-called intermediate two-dimensional “sliding layer structure” is found^32–35^.

## Discussion

In our analysis of the OASIS data, the appearance and disappearance of a quasi-long-range hexagonal lattice, formed by isotropic droplets on a spherical bubble, was observed. The driving force for such “time crystalline” behavior is hydrodynamic flow with non-uniform velocity (not reducing to a simple planar shear flow) within the Sm*A* film forming the surface of the bubble. It is worth mentioning here another avenue of research related to this work, namely colloidal systems deformable by external stresses (see e.g.,^36–41^). In such conditions, disordered colloidal particles can be dynamically forced to order in space (crystallize) or, conversely, initially ordered particles can be melted under the influence of shear stresses. These investigations enable the measurement of the shear moduli and the mean-squared displacements of the colloidal particles. Then one can apply the well-known Lindemann criterion (F. Lindemann, Z. Phys, **11**, 609, (1910)) to analyze melting and freezing phenomena and non-equilibrium phase coexistence (which occurs since the shear flow could locally increase the particle volume fraction beyond the liquid–solid phase transition). Applying shear stresses to relatively soft (although often made from hard spheres) colloidal structures is one of the most successful methods employed to deform and orient anisotropic phases. When applying a shear field, the system is transferred from equilibrium into a non-equilibrium state. Initiated by the shear, orientation arises from the complex interplay between structure and flow. Little is known about the nature of the non-equilibrium state that depends on the viscoelastic properties of the material. Depending on the strain amplitude (and frequency in the case of oscillatory shear experiments), applied shear can drive a system far from its original equilibrium state, where the measurable rheological parameters show strongly non-linear behavior. The behavior strongly depends on the ratio of relaxation times of positional and orientational fluctuations and applied non-stationary shear rates. If the characteristic relaxation time is shorter than the shear rate, the flow merely translates fluctuations in space. In the opposite limit, when the relaxation time for those fluctuations exceeds the characteristic time of the shear flow, fluctuations live in an averaged environment, similar to the equilibrium one. For a stationary shear, one should not expect occurrence of new (not observed in equilibrium) structures. In principle, many ideas from this field might be applied to other systems although their practical implementation requires much additional work beyond the scope of this paper. Although Fourier transforms are useful in analyzing the dynamics of ideal periodic structures which change in time (see, for example^42^), in the case of our experiments, where there is only quasi-periodicity along the rows and the number of rows is limited, this technique is not as reliable as directly using particle tracking data to extract information about spatial correlations in the observed structures.

Microgravity is essential for our experiments. Under normal gravity, a number of difficulties have to be overcome to realize a similar experiment even in flat free-standing film geometry. A major problem is that the film has to be heated to a well-defined temperature and kept at that temperature. Even small gradients of the temperature profile lead to thermally driven convection in the film that advects the liquid crystal material and affects the subtle interactions stabilizing the lattices. Moreover, the meniscus of a flat film has a strong influence on liquid inclusions in the film. The effects of the meniscus are minimized in tethered bubbles but performing observations of inclusions on spherical smectic bubbles in normal gravity is practically impossible since a fast sedimentation of the droplets occurs.

In summary, two-dimensional structures of droplets in smectic films were observed under microgravity conditions. A stationary hexagonal arrangement of droplets was observed and analyzed quantitatively. When the droplets in the film move with a velocity gradient, the appearance and disappearance of the hexagonal structure was observed. Such behavior can be considered as a dynamic analogue of crystalline translation order.

The observed behavior in the OASIS experiments resembles a kind of time crystal. The structures are time dependent, appearing at certain moments of time in a non-equilibrium dynamical system. This is the characteristic feature of the phenomenon called “time crystals” in modern literature. One of the aims of our work is to show that liquid crystals can serve to model or simulate such non-trivial behavior. At this time, a unified theory explaining the stability of the hexagonal structure in the absence of in-plane orientational interactions is still not available. The necessary conditions for creating structures of droplets with different types of spatio-temporal ordering will be explored in future experiments.

## Methods

The experiment was performed in microgravity conditions of the International Space Station (ISS) as a part of OASIS mission^1^. The complete setup is enclosed in a thermostated chamber for the control of the sample temperature. Smectic bubbles are inflated automatically on the tip of two coaxial capillaries. The gap between inner and outer capillaries is filled with smectic material. By pumping a small amount of the mesogen from a reservoir to the tip, we first create a smectic cap above the inner capillary. Then, by slowly pumping air through the inner capillary, an air bubble topped by a freely suspended smectic film forms. As soon as this film has formed, more air is pumped in so that a smectic bubble of the desired size (approximately 1.5 cm diameter) forms. Limited control of the film thickness of this bubble can be achieved by varying the initial inflation speed. When a small bubble with fairly uniform film thickness has formed, further inflation usually preserves this thickness. Smectic film material is constantly supplied from the meniscus at the capillary. Film thicknesses in this experiment were of the order of a few dozen to a few hundred nanometers. After the desired bubble size was reached, we heated the chamber at the highest achievable heating rate to close to the smectic A to isotropic transition. A slight overshoot of a few hundred mK, or perhaps up to about 1 K, led to partial melting of the inner layers of the smectic film. The temperature was then kept in the vicinity of the bulk clearing temperature (which is a few degrees below the film melting point). The molten smectic material organizes in droplets with a fairly monodisperse size distribution. As long as the temperature is kept constant, the number of droplets and their sizes remains unchanged at least within the experiment time of several minutes. Airflow was generated near the bubble surface using four airjet needles. These needles were multifunctional and were also used for local heating and for applying electric fields. Airflow was used following creation of the bubble in order to generate island emulsions and in order to rotate the bubble so that the micro-view camera could observe different regions of the bubble surface. In the experiments analyzed here, airflow was employed to induce shear flow in the bubble.

## References

[1] Clark NA, Eremin A, Glaser MA, Hall N, Harth K, Klopp C, Maclennan JE, Park CS, Stannarius R, Tin P, Thurmes WN, Trittel T (2017). Realization of hydrodynamic experiments on quasi-2D liquid crystal films in microgravity. Adv. Space Res..

[2] Klopp C, Trittel T, Eremin A, Harth K, Stannarius R, Park CS, Maclennan JE, Clark NA (2019). Structure and dynamics of a two-dimensional colloid of liquid droplets. Soft Matter.

[3] Dolganov PV, Cluzeau P, Dolganov VK (2019). Interaction and self-organization of inclusions in two-dimensional free-standing smectic films. Liq. Cryst. Rev..

[4] Cluzeau P, Poulin P, Joly G, Nguyen HT (2001). Interactions between colloidal inclusions in two-dimensional smectic-*C** films. Phys. Rev. E.

[5] Völtz C, Stannarius R (2005). Buckling instability of droplet chains in freely suspended smectic films. Phys. Rev. E.

[6] Völtz C, Stannarius R (2004). Self-organization of isotropic droplets in smectic-C free-standing films. Phys. Rev. E.

[7] Dolganov PV, Dolganov VK (2006). Director configuration and self-organization of inclusions in two-dimensional smectic membranes. Phys. Rev. E.

[8] Dolganov PV, Cluzeau P (2008). Influence of chirality on director configuration and droplet interaction in ferroelectric free-standing films. Phys. Rev. E.

[9] Qi Z, Park CS, Glaser MA, Maclennan JE, Clark NA (2016). Experimental realization of an incompressible Newtonian fluid in two dimensions. Phys. Rev. E.

[10] Radzihovsky SP, Cranfill C, Nguyen Z, Park CS, Maclennan JE, Glaser MA, Clark NA (2017). Two-dimensional island emulsions in ultrathin, freely-suspended smectic liquid crystal films. Soft Matter.

[11] Pettey D, Lubensky TC, Link DR (1998). Topological inclusions in 2D smectic C films. Liq. Cryst..

[12] Dolganov PV, Demikhov EI, Dolganov VK, Bolotin BM, Krohn K (2003). Collective behavior of light-induced droplets in smectic membranes. Eur. Phys. J. E.

[13] Wilczek F (2012). Quantum time crystals. Phys. Rev. Lett..

[14] Shapere A, Wilczek F (2012). Classical time crystals. Phys. Rev. Lett..

[15] Bruno P (2013). Impossibility of spontaneously rotating time crystals: A No-Go theorem. Phys. Rev. Lett..

[16] Kozin VK, Kyriienko O (2019). Quantum time crystals from hamiltonians with long-range interactions. Phys. Rev. Lett..

[17] M. Kleman, O. Lavrentovich, *Soft Matter Physics: An Introduction.* Springer, New York (2003). Pattern Formation in Liquid Crystals. In (ed. Buka, A. & Kramer, L.) (Springer, New York, 1996).

[18] Boyle L, Khoo JY, Smith K (2016). Symmetric satellite swarms and choreographic crystals. Phys. Rev. Lett..

[19] Libal A, Balazs T, Reichhardt C, Reichhardt CJO (2020). Colloidal dynamics on a choreographic time crystal. Phys. Rev. Lett..

[20] Kibble TWB (1976). Topology of cosmic domains and strings. J. Phys. A Gen. Phys..

[21] Khemani V, Lazarides A, Moessner R, Sondhi SL (2016). Phase Structure of driven quantum systems. Phys. Rev. Lett..

[22] von Keyserlingk CW, Khemani V, Sondhi SL (2016). Absolute stability and spatiotemporal long-range order in floquet systems. Phys. Rev. B.

[23] Else DV, Bauer B, Nayak C (2016). Floquet time crystals. Phys. Rev. Lett..

[24] Zhang J, Hess PW, Kyprianidis A, Becker P, Lee A, Smith J, Pagano G, Potirniche I-D, Potter AC, Vishwanath A, Yao NY, Monroe C (2017). Observation of a discrete time crystal. Nature.

[25] Choi S, Choi J, Landig R, Kucsko G, Zhou H, Isoya J, Jelezko F, Onoda S, Sumiya H, Khemani V, von Keyserlingk C, Yao NY, Demler E, Lukin MD (2017). Observation of discrete time-crystalline order in a disordered dipolar many-body system. Nature.

[26] Brock JD, Birgeneau RJ, Litster D, Aharony A (1989). Hexatic ordering in liquid crystal films. Contemp. Phys..

[27] Zaluzhnyy IA, Kurta RP, Sulyanova EA, Gorobtsov OYu, Shabalin AG, Zozulya AV, Menushenkov AP, Sprung M, Krowczynski A, Gorecka E, Ostrovskii BI, Vartanyants IA (2017). Structural studies of the bond-orientational order and hexatic-smectic transition in liquid crystals of various compositions. Soft Matter.

[28] Crandall RS, William R (1980). Crystallization of electrons on the surface of liquid helium. Phys. Lett. A.

[29] Chaikin P, Lubensky T (1995). Principles of Condensed Matter Physics.

[30] Marrink SJ, Risselada J, Mark AE (2005). Simulation of gel phase formation and melting in lipid bilayers using a coarse grained model. Chem. Phys. Lipids.

[31] Petrov OF, Vasiliev MM, Vaulina OS, Stacenko KB, Vasilieva EV, Lisin EA, Tun Y, Fortov VE (2015). Solid-hexatic-liquid transition in a two-dimensional system of charged dust particles. EPL.

[32] Ackerson BJ, Clark NA (1981). Shear-Induced Melting. Phys. Rev. Lett..

[33] Ackerson BJ, Clark NA (1984). Shear-induced partial translational ordering of a colloidal solid. Phys. Rev. A.

[34] Ackerson BJ, Hayter JB, Clark NA, Cotter L (1986). Neutron scattering from charge stabilized suspensions undergoing shear. J. Chem. Phys..

[35] Lopez-Barron CR, Wagner NJ, Porcar L (2015). Layering, melting, and recrystallization of a close-packed micellar crystal under steady and large-amplitude oscillatory shear flows. J. Rheol..

[36] Imhof A, van Blaaderen A, Dhont JKG (1994). Shear melting of colloidal crystals of charged spheres studied with rheology and polarizing microscopy. Langmuir.

[37] Amos RM, Rarity JG, Tapster PR, Shepherd TJ, Kitson SC (2000). Fabrication of large-area face-centered-cubic hard-sphere colloidal crystals by shear alignment. Phys. Rev. E.

[38] Wille A, Valmont F, Zahn K, Maret G (2002). Shear modulus of two-dimensional colloidal crystals. Europhys. Lett..

[39] Derks D, Wu YL, van Blaaderen A, Imhof A (2009). Dynamics of colloidal crystals in shear flow. Soft Matter.

[40] Dullens RPA, Bechinger C (2011). Shear thinning and local melting of colloidal crystals. Phys. Rev. Lett..

[41] Struth B, Hyun K, Kats E, Meins T, Walter M, Wilhelm M, Grübel G (2011). Observation of new states of liquid crystal 8CB under nonlinear shear conditions as observed via a novel and unique rheology/small-angle X-ray scattering combination. Langmuir.

[42] Perinet N, Juric D, Tuckerman LS (2012). Alternating hexagonal and striped patterns in faraday surface waves. Phys. Rev. Lett..

